# Advisory Committee on Immunization Practices Recommended Immunization Schedule for Adults Aged 19 Years or Older — United States, 2025

**DOI:** 10.15585/mmwr.mm7402a3

**Published:** 2025-01-16

**Authors:** A. Patricia Wodi, Anindita N. Issa, Charlotte A. Moser, Sybil Cineas

**Affiliations:** ^1^Immunization Services Division, National Center for Immunization and Respiratory Diseases, CDC; ^2^Vaccine Education Center, Children's Hospital of Philadelphia, Philadelphia, Pennsylvania; ^3^The Warren Alpert Medical School of Brown University, Providence, Rhode Island.

At its October 2024 meeting, the Advisory Committee on Immunization Practices* (ACIP) approved the Recommended Immunization Schedule for Adults Ages 19 Years or Older, United States, 2025. The schedule supports health care providers, as well as public health and other professionals, by providing a consolidated summary of current ACIP recommendations for adult vaccination. The 2025 schedule includes several updates to the cover page, tables, notes, and appendix.^†^ The addendum remains part of the schedule and will be used to summarize new or updated ACIP recommendations that occur before the next annual schedule update. Health care providers are strongly encouraged to use all parts of the schedule (the cover page, tables, notes, appendix, and addendum) together when making recommendations for individual patients. The 2025 adult immunization schedule can be found on the CDC website (https://www.cdc.gov/vaccines/hcp/imz-schedules/index.html).

Consistent with previous years’ schedules, the 2025 adult immunization schedule is recommended by ACIP (https://www.cdc.gov/acip/index.html) and approved by CDC (https://www.cdc.gov), the American College of Physicians (https://www.acponline.org), the American Academy of Family Physicians (https://www.aafp.org), the American College of Obstetricians and Gynecologists (https://www.acog.org), the American College of Nurse-Midwives (https://www.midwife.org), the American Academy of Physician Associates (https://www.aapa.org), the American Pharmacists Association (https://www.pharmacist.com), and the Society for Healthcare Epidemiology of America (https://shea-online.org).

ACIP’s recommendations for use of each vaccine are developed after in-depth reviews of vaccine-related data including disease epidemiology and societal impacts, vaccine efficacy and effectiveness, vaccine safety, quality of evidence, feasibility of program implementation, impact on health equity, and economic analyses of immunization policy (*1*,*2*). For each vaccine in the schedule, clinical trials are conducted in the context of standard-of-care related to the routine adult immunization schedule (*3*). Routinely recommended vaccines are monitored by CDC and the Food and Drug Administration (FDA) for safety through ongoing and cumulative efforts including multiple surveillance systems, safety studies, and review of the literature (https://www.cdc.gov/vaccine-safety-systems/about/cdc-monitoring-program.html). Recommendations for specific vaccines that occur between annual schedule updates^§^ are summarized in the addendum section; however, health care providers should refer to detailed ACIP recommendations for use of each vaccine (https://www.cdc.gov/acip-recs/hcp/vaccine-specific/index.html). ACIP vaccine recommendations do not establish mandates.

The use of vaccine trade names in this report and in the adult immunization schedule is for identification purposes only and does not imply endorsement of a specific product by ACIP or CDC.

## Changes in the 2025 Adult Immunization Schedule

Compared with the 2024 adult schedule, vaccine-specific changes in the 2025 immunization schedule for adults include new and updated recommendations for COVID-19 vaccines (*4*), influenza vaccines (*5*), meningococcal serogroup B vaccines (*6*), pneumococcal conjugate vaccines (PCV) (*7**,**8*), and respiratory syncytial virus vaccines (RSV) (*9*). In all sections of the schedule, recommended influenza vaccines have been changed from the quadrivalent to trivalent formulation to be consistent with the vaccine products approved by FDA for the 2024–25 influenza season. In addition, inactivated polio vaccine was added to the Tables. Other changes include clarification in the Notes sections for hepatitis B vaccine (HepB); mpox vaccine (Mpox); and tetanus and diphtheria toxoids, and acellular pertussis vaccine (Tdap).

### Cover Page

• Trivalent adjuvanted inactivated influenza vaccine (aIIV3), trivalent cell culture–based inactivated influenza vaccine, trivalent high-dose inactivated influenza vaccine (HD-IIV3), newly licensed 21-valent pneumococcal conjugate vaccine (PCV21), and the newly licensed mRNA respiratory syncytial virus vaccine (mResvia) were added to the table listing abbreviations and trade names of the vaccines.

### Table 1 (Age-Based Immunization Schedule)

• The legend definition for the gray box was revised to harmonize with Table 2 and the child and adolescent immunization schedule. The text states, “No Guidance/Not Applicable.”

• **COVID-19 row:** The text overlay was revised to reflect updated vaccination recommendations. The text overlay for adults aged 19–64 years now states, “1 or more doses of updated 2024–2025 vaccine (See Notes),” and that for those aged ≥65 years states, “2 or more doses of updated 2024–2025 vaccine (See Notes).”

• **Influenza row:** This row was revised to reflect the preferential recommendation for use of HD-IIV3, aIIV3, and trivalent recombinant influenza vaccine in persons aged ≥65 years. In addition, a purple row and overlaying text is used to reflect the recommendation adding HD-IIV3 and aIIV3 to the vaccines that may be administered to solid organ transplant recipients aged 19–64 years who are receiving immunosuppressive medications.

• **IPV row:** This row is a new addition to the table. The color of this row is yellow, indicating that vaccination is routinely recommended for all adults who are incompletely vaccinated. The text overlay states, “Complete 3-dose series if incompletely vaccinated. Self-report of previous doses acceptable (See Notes).”

• **Mpox row:** The text overlay “2 doses” was added.

• **Pneumococcal row:** PCV21 was added to the list of recommended pneumococcal conjugate vaccines. For adults aged ≥50 years, the row is yellow, indicating that pneumococcal vaccination is universally recommended for adults in this age group if they have never received a dose of PCV (PCV15, PCV20, or PCV21) or if their previous pneumococcal vaccination history is unknown. For adults aged 19–49 years, the row is purple, indicating that pneumococcal vaccination is recommended for adults in this age group if they have medical conditions or other risk factors that increase their risk for pneumococcal disease.

• **RSV row:** This row was revised to reflect current RSV recommendations for adults aged ≥60 years. For adults aged ≥75 years, the row is yellow, indicating that vaccination is universally recommended for adults in this age group if they have not been previously vaccinated. For adults aged 60–74 years, the row is purple, indicating that vaccination is recommended for this age group if they have a risk factor or other indication that increases their risk for severe RSV disease.

### Table 2 (Immunization Schedule by Medical Indication)

• **COVID-19 row:** In the column for immunocompromised persons (excluding those with HIV infection) and in the column for those with HIV infection and CD4+ T-lymphocyte count <15% or <200/mm^3^, the row color was changed to brown to reflect that additional doses are recommended.

• **Influenza (inactivated, recombinant) row:** A text overlay “Solid organ transplant (See Notes)” was added under the immunocompromised (excluding HIV) column to reflect updated vaccination recommendations for this subgroup.

• **IPV row:** This row is a new addition to the table; it includes an orange bar for the pregnancy column, indicating that vaccination might be indicated if benefit of protection outweighs the risk for an adverse reaction. For other columns, the row is yellow, indicating that vaccination is routinely recommended for all adults who are incompletely vaccinated. The text overlay states, “Complete 3-dose series if incompletely vaccinated. Self-report of previous doses acceptable (See Notes).”

• **RSV row:** This row was revised to reflect current RSV recommendations. Except for the pregnancy column, all other columns are purple indicating vaccination is recommended for some adults who have these conditions. The text overlay “See Notes” is added to medical conditions known to increase risk for severe RSV disease.

### Vaccine Notes

The notes for each vaccine are presented in alphabetical order. Edits have been made throughout the Notes section to harmonize language, to the greatest extent possible, with that in the child and adolescent immunization schedule.

• **COVID-19:** The “Routine vaccination” and “Special situations” sections were revised to reflect recommendations for use of 2024–2025 COVID-19 vaccine in adults. The “Routine vaccination” section describes recommendations for the general population, and the “Special situations” section describes recommendations for persons who are moderately or severely immunocompromised. In each section, the recommendations are outlined by previous COVID-19 vaccination history, and in the “Routine vaccination” section, they are also outlined by age group. Hyperlinks to the interim clinical considerations for use of COVID-19 vaccines as well as Emergency Use Authorization indications for COVID-19 vaccines are included.

• **HepB:** In the “Special situations” section, dosing recommendations for immunocompromised persons aged ≥20 years were added. The guidance on vaccines that are not recommended for use during pregnancy was revised to remove Heplisav-B.

• **Influenza:** The “Routine vaccination” section was updated with new recommendations adding aIIV3 and HD-IIV3 as vaccine options that can be administered to solid organ transplant recipients aged 19–64 years who are receiving immunosuppressive medications.

• **Meningococcal:** The “Special situations” section for MenACWY was revised to clarify that booster doses are recommended after completion of the primary series. In the MenB notes, both the “Routine vaccination” and “Special situations” sections were revised to include the new Bexsero vaccination schedule. For healthy persons aged 16–23 years, a series of 2 doses separated by 6 months is recommended based on shared clinical decision-making. Adults at increased risk for serogroup B meningococcal disease are recommended to receive a 3-dose series at 0-, 1–2-, and 6-month intervals. In addition, the information for MenB use during pregnancy was revised to clarify that the recommendation to delay vaccination until after pregnancy is based on a lack of safety data in pregnant persons.

• **Mpox:** Language for vaccinating health care personnel was revised to clarify that vaccination to protect against occupational risk in health care settings is not routinely recommended.

• **Pneumococcal:** PCV21 was added to all sections of the notes as an option when vaccination is indicated. The “Routine vaccination” section now reflects the new recommendation for universal vaccination for adults aged ≥50 years, and the “Special situations” section outlines the risk-based recommendation for adults aged 19–49 years. In addition, information was added for use of pneumococcal vaccines during pregnancy, and recommendations for situations when PPSV23 is unavailable.

• **RSV:** The “Routine vaccination” section now outlines recommendations for universal vaccination for pregnant persons and adults aged ≥75 years. The “Special situations” section includes risk-based recommendations for adults aged 60–74 years and the list of medical and other conditions that increase the risk for severe RSV disease. Language was added to clarify that persons can self-attest to the presence of a risk factor.

• **Tdap:** The “Routine vaccination” section was revised to describe the recommendations according to previous vaccination history.

### Appendix (Contraindications and Precautions)

• **Hepatitis B row:** In the “Contraindicated and Not Recommended” column, the language about vaccines not recommended for use during pregnancy was revised to remove Heplisav-B. The corresponding footnote with hyperlink to the pregnancy registries was also revised to remove information for the Heplisav-B registry, which is no longer active.

• **Pneumococcal row:** PCV21 was added.

• **Zoster row:** The “Precautions” column was revised to clarify that vaccination should be delayed during a current episode of herpes zoster.

### Additional Information

The Recommended Adult Immunization Schedule, United States, 2025, is available at https://www.cdc.gov/vaccines/hcp/imz-schedules/adult-age.html. The full ACIP recommendations for each vaccine are also available at https://www.cdc.gov/acip-recs/hcp/vaccine-specific/index.html. All vaccines identified in Tables 1 and 2 (except Zoster vaccine) also appear in the Recommended Immunization Schedule for Children and Adolescents, United States, 2025 (https://www.cdc.gov/vaccines/hcp/imz-schedules/child-adolescent-age.html). For vaccines that appear in both the adult immunization schedule and the child and adolescent immunization schedule, the language in both schedules has been harmonized to the greatest extent possible.

## Footnotes

* Recommendations for routine use of vaccines in adults are developed by ACIP, a federal advisory committee chartered to provide expert external advice and guidance to the CDC director on use of vaccines and related agents for the control of vaccine-preventable diseases in the civilian population of the United States. Recommendations for routine use of vaccines in adults are harmonized to the greatest extent possible with recommendations made by the American Academy of Pediatrics, the American Academy of Family Physicians, and the American College of Obstetricians and Gynecologists. ACIP recommendations become official agency guidelines once the recommendations have been adopted by the CDC director. Additional information about ACIP is available at https://www.cdc.gov/acip/index.html.

† Past immunization schedules are available at https://www.cdc.gov/vaccines/hcp/imz-schedules/resources.html.

§ CDC encourages organizations to use syndication as a more reliable method for displaying the most current and accurate immunization schedules on an organization’s website, rather than copying these schedules to their websites. Use of content syndication requires a one-time step that ensures an organization’s website displays current schedules as soon as they are published or revised; instructions for the syndication code are available on CDC’s website (https://www.cdc.gov/vaccines/hcp/imz-schedules/syndicate-resources.html). CDC also offers technical assistance for implementing this form of content syndication (requests can be emailed to ncirdwebteam@cdc.gov).
